# GNAI2 Promotes Proliferation and Decreases Apoptosis in Rabbit Melanocytes

**DOI:** 10.3390/genes12081130

**Published:** 2021-07-25

**Authors:** Shuaishuai Hu, Yingying Dai, Shaocheng Bai, Bohao Zhao, Xinsheng Wu, Yang Chen

**Affiliations:** 1 College of Animal Science and Technology, Yangzhou University, Yangzhou 225009, China; 18852726848@163.com (S.H.); d13921869520@163.com (Y.D.); bsc305972572@163.com (S.B.); 007623@yzu.edu.cn (B.Z.); yangc@yzu.edu.cn (Y.C.); 2 Joint International Research Laboratory of Agriculture & Agri-Product Safety, Yangzhou University, Hangzhou 310021, China

**Keywords:** GNAI2, melanocyte, proliferation, apoptosis

## Abstract

GNAI2 (G protein subunit alpha i2) is a signaling modulator or transducer, involved in several transmembrane signaling systems, that plays a vital role in the melanogenesis signaling pathway. However, whether GNAI2 regulates cell proliferation and apoptosis in rabbit melanocytes is not known. We found that GNAI2 was differentially expressed in rabbits with different coat colors using qRT-PCR and Wes assays. Furthermore, it was observed that the rabbits with black skin had the highest GNAI2 levels, and those with white skin had the lowest expression. The coding sequence of GNAI2 was successfully cloned and inserted into pcDNA3.1 and pcDNA3.1-Myc vectors. It was observed that the GNAI2 protein was mainly localized in the cytoplasm using the indirect immunofluorescence staining assay. Overexpression of GNAI2 significantly increased melanin content, promoted melanocyte proliferation, and inhibited melanocyte apoptosis. On the contrary, the knockdown of GNAI2 using siRNA had the opposite effect. In addition, GNAI2 significantly increased the mRNA expression levels of the melanin-related genes TYR, GPNMB, PMEL, and DCT in rabbit melanocytes. The results suggested that GNAI2 regulated melanocyte development by promoting melanocyte proliferation and inhibiting apoptosis.

## 1. Introduction

Melanogenesis is a complex process of the synthesis and storage of melanin initiated by tyrosinase, which is synthesized by melanocytes, after it enters the melanosomes [1]. Melanogenesis plays an important role in the formation of different coat colors in mammals. There are two distinct types of melanin pigments: eumelanin and pheomelanin, the proportions of which determine the coat color of the animal [2,3,4]. The difference in the eumelanin content leads to the formation of different hair types in humans, such as black, brown, light brown, and blond [5]. Previous studies have found that there is lower eumelanin content in those with lighter skin, which is highly sensitive to UV exposure, compared to those with darker skin [6]. Melanogenesis is regulated by several signaling systems, transcription factors, and melanin-related genes [7,8]. MC1R regulates melanogenesis andt was significantly decreased in the skin of a leucistic and blind cypriniform fish (O. daqikongensis, Nemacheilidae) [9]. α-MSH plays an important role in melanogenesis by regulating the proportions of pheomelanin and eumelanin via MC1R [10]. The TYR gene is essential for melanin biosynthesis in melanocytes; it was differentially expressed in black and white feather bulbs, indicating that it plays an important role in the melanin formation of feather bulb color in the plumage [11]. Several studies have suggested that the expression of TYR was related to the content of eumelanin; the overexpression of TYR could increase eumelanin content in mammals [12]. Eumelanin content was related to TYR activity. Previous studies have demonstrated that TYR activity in black bone sheep was significantly higher than that in non-black bone sheep [13].

GNAI2 (G protein subunit alpha i2), belonging to the G protein family, is one of the key genes involved in melanogenesis. G proteins consist of three subunits, α, β, and γ, and are signal transducers, which connect receptors to effectors in order to regulate intracellular signaling [14]. G protein-coupled receptors (GPCRs) receive external signals that activate the G proteins to transmit the signal to further regulate the growth and development of the organisms [15]. At present, a large number of G proteins are identified, including Gs, Gt, Gi, and Go [16]. Previous research shows that GNAI2 is mostly involved in cell injury and inflammatory response [17], tumorigenesis [18], hepatic ischemia-reperfusion injury [19], etc. GNAI2 is mainly expressed in immune cells and plays an essential role in regulating cellular viability and migration [20]. In addition, previous studies have found that GNAI2 associated with melanogenesis was identified in the growth process of brindle cattle [21]. However, it remains unclear whether GNAI2 is involved in the formation of rabbits’ coat colors and regulates rabbit melanocyte proliferation and apoptosis.

The purpose of the present research is to explore the molecular function of GNAI2 in melanogenesis, and melanocyte proliferation and apoptosis after GNAI2 overexpression and knockdown. The study could provide an important theoretical basis for the molecular mechanism of melanogenesis to regulate the formation of mammalian coat colors.

## 2. Materials and Methods

### 2.1. Ethics Statement

This study was carried out in accordance with the recommendations of Jiangsu Administration Rule of Laboratory Animals and strictly followed Institutional Animal Care and Maintenance protocols. 

### 2.2. Experimental Animals

A total of 18 adult Rex rabbits of different coat colors (black, white, chinchilla, brown, gray, and gray-yellow) were provided by Zhejiang Yuyao Xinnong Rabbit Industry Co., Ltd (Yuyao, Zhejiang, China). Three biological rabbits of each of the coat colors were used. The dorsal skin of Rex rabbits (1.5 cm × 1.5 cm) was collected for RNA and protein extraction to determine the GNAI2 expression in the different coat colors.

### 2.3. Overexpression Vector Construction

The coding sequence (CDS) of GNAI2 (NCBI Reference Sequence: XM_008260677.2) was cloned using rabbit melanocyte cDNA as a template using ClonExpress II One Step cloning kit (Vazyme, Nanjing, China). The GNAI2 CDS was inserted into pcDNA3.1 and pcDNA3.1-Myc vectors using restriction enzymes EcoRI and XbaI. The primers used are shown in Table 1.

### 2.4. Cell Culture and Transfection

Melanocyte culture was established using the method described by Chen et al. [22]. An adult black rabbit was used to isolate the melanocytes. A tissue sample of approximately 1.5 cm × 1.5 cm was excised from the dorsal skin of the rabbit. Rabbit melanocytes were cultured in Melanocyte Medium-2 (ScienCell, San Diego, CA, USA) supplemented with 1% PMA-free melanocyte growth supplement, 0.5% fetal bovine serum (FBS), and 0.5% penicillin/streptomycin solution (ScienCell, San Diego, CA, USA). Melanocytes were transferred to 24-well plates and incubated at 37 °C with 5% CO_2_. When the culture was 70–90% confluent, overexpression and knockdown experiments were performed using 1 μg GNAI2 plasmid and 1 μL siRNAs (Table 2), respectively. Both were diluted in 25 μL Opti-MEM™ medium (Gibco, Carlsbad, CA, USA) and 2 μL diluted Lipofectamine™ 3000 (Lipofectamine™ 3000 diluted in 25 μL Opti-MEM™ medium) was added to both the mixtures. These mixtures were incubated for 10-15 min at room temperature and were then added to cultured melanocytes separately, and the cells were incubated at 37 °C with 5% CO_2_ for 48 h. 

### 2.5. Indirect Immunofluorescence Staining Assay

The cells transfected with pcDNA3.1-Myc-GNAI2 plasmid were washed in phosphate-buffered saline (PBS) (HyClone, Logan, UT, USA) three times lightly and fixed at room temperature for 20 min using cold (4%) paraformaldehyde. Then, the cells were washed again using PBS and treated with 0.2% Triton X-100 (Solarbio, Beijing, China). After 10 min, goat serum was used to block the cells for 30 min. Then, the cells were incubated using the MYC tag monoclonal antibody (Proteintech, Wuhan, China) at 4 °C overnight. Subsequently, Cy3-conjugated Affinipure Goat Anti-Mouse IgG (H+L) (Proteintech, Wuhan, China) was added and the cells were incubated at room temperature for 2 h in dark. DAPI staining solution was added to the cells and they were incubated for 10 min. The cells were observed using a fluorescence inverted microscope. 

### 2.6. Quantitative Real-Time Polymerase Chain Reaction (qRT-PCR) Analysis

Total RNA from the rabbit melanocytes was isolated using RNAsimple Total RNA kit (Tiangen Biotech Co., Ltd., Beijing, China). Total RNA was reverse-transcribed using HiScript III 1st Strand cDNA Synthesis Kit (+gDNA wiper) (Vazyme, Nanjing, China). Quantitative real-time PCR was performed using ChamQTM SYBR^®^ qPCR Master Mix (Vazyme, Nanjing, China) in QuantStudio^®^5 (Applied Biosystems; Thermo Fisher Scientific, Foster City, CA, USA). The relative mRNA expression was calculated using the 2^−ΔΔCt^ method [23]. The primers used are shown in Table 3.

### 2.7. Protein Preparation and Western Blotting

Rabbit melanocyte lysates were obtained using cell lysis buffer (Beyotime, Shanghai, China) containing 1% phenylmethanesulfonylfluoride (PMSF). Protein concentration was estimated using the BCA protein assay kit (Beyotime, Shanghai, China). Protein assay was performed using Wes’s automated Western blotting system (Wes assay) [24] according to the manufacturer’s instructions. GAPDH monoclonal antibodies and GNAI2 monoclonal antibodies (Proteintech, Wuhan, China) were used.

### 2.8. Melanin Content Measurement

GNAI2 overexpression and knockdown experiments were performed using melanocytes. Cells were collected 72 h after transfection. The cells were lysed using 1 M NaOH and the lysate was incubated at 80 °C for 1 h. Optical density (OD) was measured at 475 nm using Infinite M200 PRO (Tecan, Männedorf, Switzerland) spectrophotometer. 

### 2.9. Cell Proliferation Assay 

Transfected cells were harvested and seeded into 96-well plates equally. Cell proliferation was detected at 0, 24, 48, and 72 h using Cell Counting Kit-8 assay (Vazyme, Nanjing, China).

### 2.10. Apoptosis Assay

Melanocytes were transfected in 6-well plates. After 48 h, cells were harvested. Apoptosis assay was performed in order to detect the level of apoptosis using an Annexin V-FITC apoptosis detection kit (Vazyme, Nanjing, China). Later, the cells were sorted using a fluorescence-activated cell sorter (FACSAria SORP flow cytometer, Becton Dickinson, San Jose, CA, USA).

### 2.11. Statistical Analysis

All statistical analyses were carried out using SPSS version 25 (SPSS Inc., Chicago, IL, USA). One-way ANOVA was employed to analyze significant differences among the study groups. All values are presented as mean ± standard deviation (SD).

## 3. Results

### 3.1. GNAI2 Was Differentially Expressed in the Skin of Rabbits with Different Coat Colors

**Figure 1:**
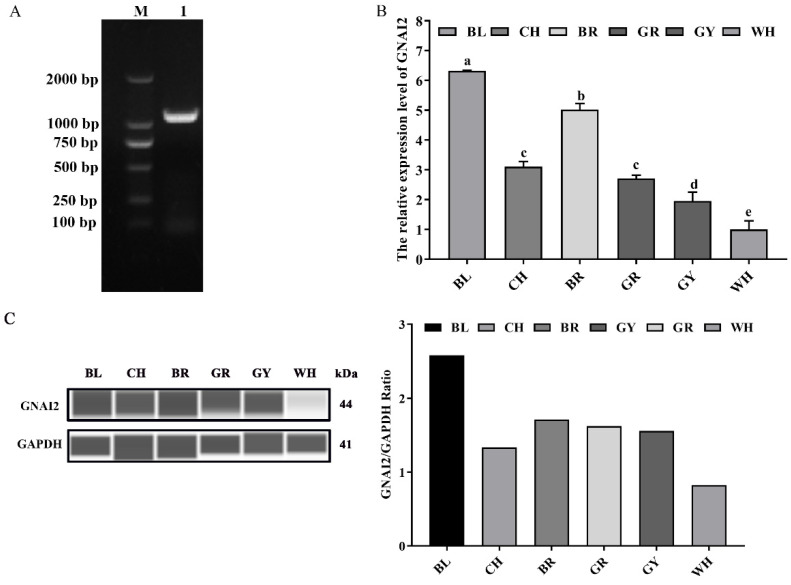
The expression levels of GNAI2 in the skin of rabbits of different coat colors: (**A**) the rabbit GNAI2 cDNA was successfully cloned. M, DL2000 DNA marker; lane 1, the coding sequence (CDS) of GNAI2; (**B**) the GNAI2 mRNA levels in the skin of rabbits of different coat colors were determined. Samples were in triplicates and the relative expression levels of the genes were determined using GAPDH as an internal control and the 2^−ΔΔCt^ method. Duncan’s Multiple Range Test was employed to compare the differences across groups. Small letters indicate significant differences among groups (*p* < 0.05); (**C**) the GNAI2 protein expression in the rabbit skin was measured using the Wes assay. Differential expression of GNAI2 in Rex rabbits with different coat colors was calculated using the relative expression ratio of GNAI2/GAPDH. BL, black; CH, chinchilla; BR, brown; GR, gray; GY, gray-yellow; WH, white.

GNAI2 was successfully cloned, and comprised 1068 bp coding 355 amino acids (Figure 1A). To determine whether GNAI2 was involved in the formation of rabbit coat colors, the expression levels of GNAI2 were detected in the skin of rabbits of different coat colors. It was found that the mRNA expression of GNAI2 in the black skin was significantly higher than that in the other skins (*p* < 0.05), and the lowest expression was detected in the white skin (Figure 1B). Similarly, GNAI2 protein levels were different in the skins of rabbits of different coat colors. The highest GNAI2 expression was found in the black skin, and the white skin had the lowest (Figure 1C).

### 3.2. GNAI2 Protein Was Mainly Localized in the Cytoplasm of Melanocytes

**Figure 2:**
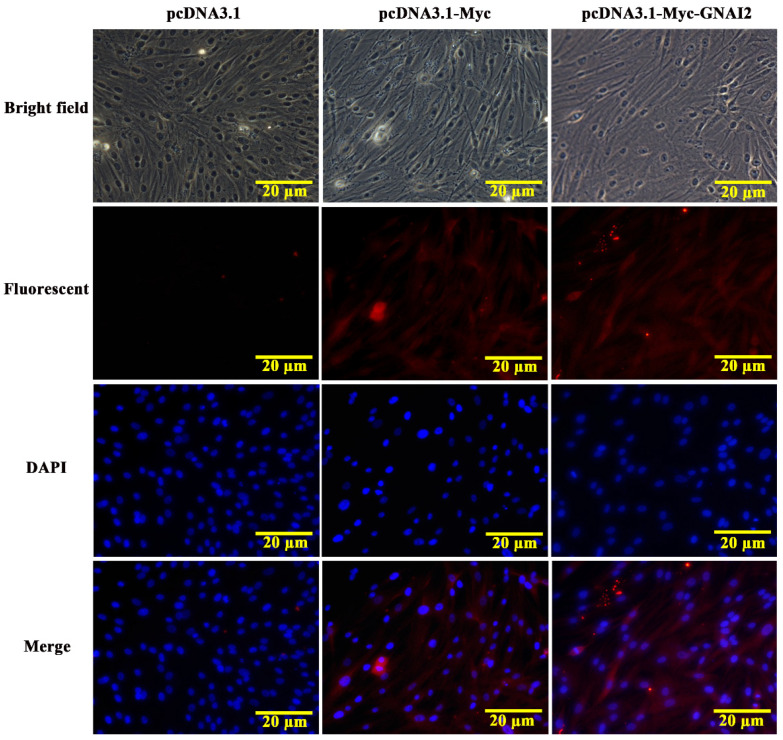
The localization of GNAI2 protein in melanocytes (400X). The subcellular localization of GNAI2 in melanocytes was determined using an indirect immunofluorescence staining assay. The pcDNA3.1 group was used as a negative control and the pcDNA3.1-Myc group was used as a positive control.

The subcellular localization of the GNAI2 protein was predicted using the protein subcellular localization prediction tool (PSORT). It was found that the GNAI2 protein was mainly expressed in the cytoplasm of melanocytes. To further verify the expression of the GNAI2 protein, an indirect immunofluorescence staining assay was performed. The pcDNA3.1 group was used as a negative control and the pcDNA3.1-Myc group was used as a positive control. It was found that the GNAI2 protein was localized in the cytoplasm of melanocytes (Figure 2), which was consistent with the prediction.

### 3.3. GNAI2 Promoted Melanogenesis

**Figure 3:**
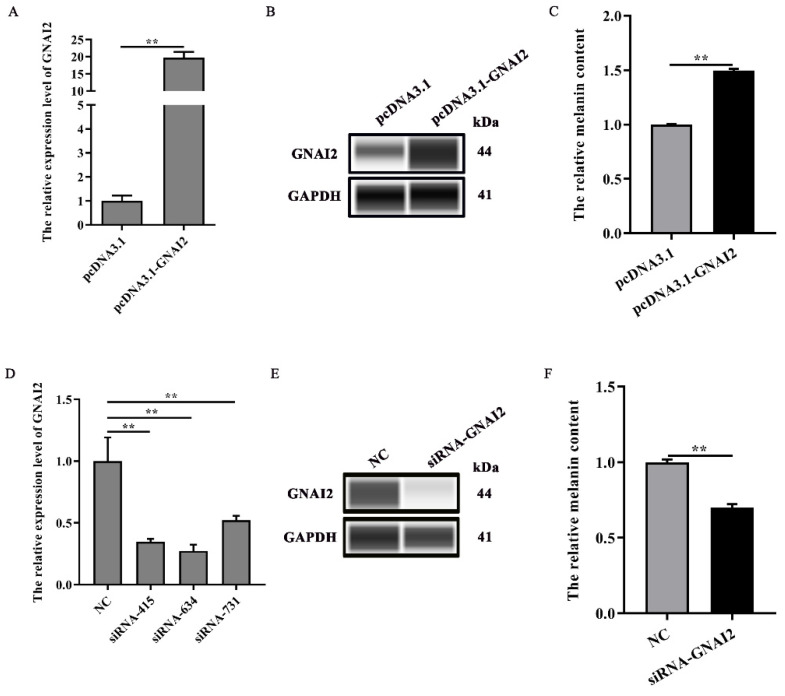
GNAI2 promoted melanogenesis: (**A**) GNAI2 mRNA expression levels in GNAI2-overexpressing melanocytes were detected using a qRT-PCR assay; (**B**) the GNAI2 protein expression was determined using Wes assay after GNAI2 was overexpressed in melanocytes; (**C**) the melanin content was measured in GNAI2-overexpressing melanocytes using NaOH lysates; (**D**) GNAI2 mRNA expression levels were determined in GNAI2-silenced melanocytes using a qRT-PCR; (**E**) the GNAI2 protein levels were determined using Wes assay after GNAI2 was knocked down in melanocytes; (**F**) the melanin content was measured in GNAI2-silenced melanocytes using NaOH lysates. **, *p* < 0.01.

Our results suggested that GNAI2 is involved in the formation of coat colors in rabbits. To confirm its role in melanogenesis, melanin content was measured when GNAI2 was overexpressed and knocked down in melanocytes. We found that GNAI2 mRNA and protein expression increased when GNAI2 was overexpressed (*p* < 0.01, Figure 3A,B). Similarly, melanin content also increased significantly (*p* < 0.01, Figure 3C). GNAI2 knockdown was performed using siRNAs. The three different siRNAs used decreased GNAI2 expression significantly (*p* < 0.01), but siRNA-634 showed the greatest effect (Figure 3D). When GNAI2 was downregulated using siRNA-634, GNAI2 protein expression (Figure 3E) and melanin content significantly decreased (*p* < 0.01, Figure 3F). 

### 3.4. GNAI2 Improved Melanocyte Proliferation and Apoptosis

**Figure 4:**
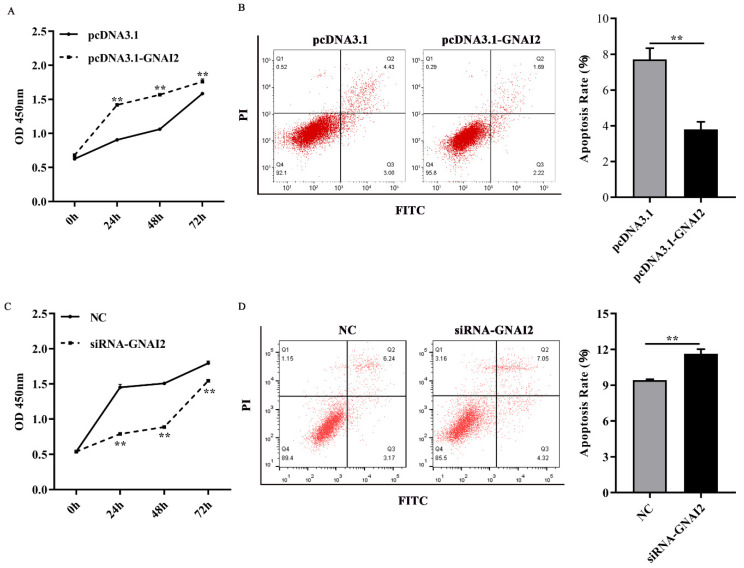
GNAI2 regulated melanocyte proliferation and apoptosis: (**A**) melanocyte proliferation was estimated using CCK-8 assay at 24, 48, and 72 h in GNAI2-overexpressing melanocytes; (**B**) melanocyte apoptosis was detected in GNAI2-overexpressing melanocytes and cellular apoptosis rate was calculated; (**C**) melanocyte proliferation was estimated in GNAI2-silenced melanocytes at 24, 48, and 72 h using the CCK-8 assay; (**D**) cellular apoptosis of GNAI2-silenced melanocytes was measured and cellular apoptosis rate was calculated. Samples were in triplicates. **, *p* < 0.01.

To further elucidate the regulatory effect of GNAI2 on melanocytes, cell proliferation and apoptosis were estimated using a CCK-8 assay and fluorescence-activated cell sorting, respectively. We found that GNAI2 promoted melanocyte proliferation and inhibited apoptosis when GNAI2 was overexpressed (Figure 4A,B). Conversely, melanocyte proliferation was inhibited and cell apoptosis was promoted when GNAI2 was downregulated (Figure 4C,D). The results demonstrated that GNAI2 promoted melanocyte proliferation and decreased apoptosis.

### 3.5. GNAI2 Overexpression and Knockdown Regulated the Expression of Melanin-Related Genes

**Figure 5:**
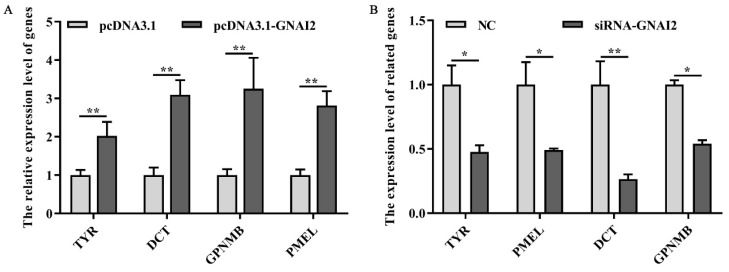
GNAI2 affected the expression of the downstream melanin-related genes: (**A**) qRT-PCR was performed to detect the mRNA expression levels of melanin-related genes in GNAI2-overexpressing melanocytes; (**B**) the expression of melanin-related genes was determined using a qRT-PCR after GNAI2 downregulation in melanocytes. *, *p* < 0.05; **, *p* < 0.01.

To check whether GNAI2 has an important effect on the melanogenesis pathway, the expression of melanin-related key genes was measured after GNAI2 overexpression and knockdown in melanocytes. The results showed that the mRNA levels of the related genes TYR, DCT, GPNMB, and PMEL were significantly upregulated following GNAI2 overexpression (*p* < 0.01, Figure 5A). Furthermore, the knockdown of GNAI2 using siRNA-634, which showed the highest effect, significantly decreased the mRNA levels of the melanin-related genes (*p* < 0.01, Figure 5B). The results indicated that GNAI2 promoted the expression of melanin-related genes.

## 4. Discussion

In the current study, it was demonstrated that the differential expression of GNAI2 was involved in the different coat colors of Rex rabbits. The greatest expression of GNAI2 was observed in the black skin, and the least expression was detected in the white skin. In addition, GNAI2 overexpression significantly promoted melanin production. Therefore, the results suggested that GNAI2 plays an important role in the formation of different coat colors in rabbits by regulating melanogenesis. Previous research demonstrated that MITF-M mRNA levels were the lowest when the C57BL/6J black mice overexpressed miR-137, and the overexpression of MITF-M increased the melanin content [25]. Melanin content in the skin of Rex rabbits of different coat colors was distinct, and the highest content was observed in those having black skin and the lowest content in those having white skin [26]. Some studies also found that Slc7a11 mRNA and protein levels were different in the skin of rabbits of different coat colors, and the rabbits that had black skin had significantly higher levels than those that had other coat colors [22]. Similarly, the expression pattern of KIT, a key proto-oncogene, was consistent with the Slc7a11 expression [27], and the difference among coat colors of Liaoning Cashmere goats could be explained by mutations in KIT [28]. These findings may provide reasonable evidence to suggest the importance of different genes in regulating the coat color in mammals.

Cell proliferation and apoptosis are the basic phenomena that help to maintain the number of cells in the body during the development and regeneration of the organism and/or its tissues. Melanocyte proliferation and apoptosis could be regulated by some key genes of the melanogenesis pathway. Previous studies indicated that Wnt5a acted as a negative regulatory gene and inhibited mouse melanocyte proliferation, and, thereby, melanogenesis [29]. Skin melanocyte proliferation was regulated by Sox10 by activating the DNA replication licensing factor MCM5 [30]. MicroRNA-143-5p regulated alpaca melanocyte migration and proliferation and melanogenesis [31]. In addition, Opsin 3 (OPN3), belonging to the G protein-coupled receptor superfamily, played a vital role in cell survival. Upon downregulation, it induced apoptosis of the human epidermal melanocytes through calcium-dependent G protein-coupled signaling and mitochondrial pathways [32]. The results from the current study demonstrated that GNAI2 promoted melanocyte proliferation when it was upregulated in melanocytes. Furthermore, apoptosis in the melanocytes increased when GNAI2 was knocked down. 

GNAI2 played an important role in melanogenesis. It regulated the expression of melanin-related genes and affected melanin synthesis. TYR and DCT are important regulators of melanogenesis. Our results showed that the expression levels of melanin-related genes TYR, DCT, GPNMB, and PMEL were significantly increased upon GNAI2 overexpression. On the contrary, the expression levels of these genes were downregulated after GNAI2 was knocked down. These results suggested that GNAI2 promoted melanin synthesis by maintaining the expression of melanin-related genes.

## 5. Conclusions

Our study suggested that GNAI2 was involved in the formation of the coat colors in Rex rabbits, depending upon its expression levels. GNAI2 overexpression promoted melanocyte proliferation and inhibited cell apoptosis. In all, GNAI2 played a positive role in melanogenesis.

## Figures and Tables

**Table 1:** The primer sequences of GNAI2 CDS.

Primers	Sequence (5′→3′)
GNAI2-F	tagtccagtgtggtggaattcGCCACCATGGGCTGCACGGTGAGC
GNAI2-R	ttgttcgaagggccctctagaGAAGAGGCCGCAGTCCTTCA

**Table 2:** Primer sequences of the GNAI2 siRNAs.

Primers	Sequence (5′ to 3′)
Negative Control	Forward: UUCUCCGAACGUGUCACGUTT
	Reverse: ACGUGACACGUUCGGAGAATT
siRNA-GNAI2-415	Forward: GCAACCUGCAGAUUGACUUTT
	Reverse: AAGUCAAUCUGCAGGUUGCTT
siRNA-GNAI2-634	Forward: GCAUCGCACAGAGUGACUATT
	Reverse: UAGUCACUCUGUGCGAUGCTT
siRNA-GNAI2-731	Forward: CCUGCACUUCAAGAUGUUUTT
	Reverse: AAACAUCUUGAAGUGCAGGTT

**Table 3:** qRT-PCR primer sequences.

Primers	Sequence (5′→3′)	Product Length (bp)
GNAI2	Forward: ACGACTCAGCCGCCTAC	119
	Reverse: GTGCGTCTCCACGATCC	
TYR	Forward: CTCTTCTTGTTGCTGTGGG	156
	Reverse: GCTGAGTAGGTTAGGGTTTTC	
DCT (TYRP2)	Forward: ATTCTGCTGCCAATGACCC	154
	Reverse: AACGGCACCATGTTATACCTG	
PMEL	Forward: GTCAGCACCCAGCTTGTCA	130
	Reverse: GCTTCATTAGTCTGCGCCTGT	
GPNMB	Forward: TCCAGATTGCAGAAGTCCCGAT	173
	Reverse: GCAGCTCTCAGTCTCGTCCA	
GAPDH	Forward: CACCAGGGCTGCTTTTAACTCT	141
	Reverse: CTTCCCGTTCTCAGCCTTGACC	

## Data Availability

All data generated or analyzed during this study are available from the corresponding author on reasonable request.

## References

[1] Videira I.F.D.S., Moura D.F.L., Magina S. (2013). Mechanisms regulating melanogenesis. An. Bras. Dermatol..

[2] Naysmith L., Waterston K., Ha T., Flanagan N., Bisset Y., Ray A., Wakamatsu K., Ito S., Rees J.L. (2004). Quantitative measures of the effect of the melanocortin 1 receptor on human pigmentary status. J. Investig. Dermatol..

[3] Slominski A., Tobin D.J., Shibahara S., Wortsman J. (2004). Melanin pigmentation in mammalian skin and its hormonal regulation. Physiol. Rev..

[4] Ito S., Wakamatsu K., Ozeki H. (2000). Chemical analysis of melanins and its application to the study of the regulation of melanogenesis. Pigment Cell Res..

[5] Ito S., Wakamatsu K. (2003). Quantitative Analysis of Eumelanin and Pheomelanin in Humans, Mice, and Other Animals: A Comparative Review. Pigment. Cell Res..

[6] Del Bino S., Ito S., Sok J., Nakanishi Y., Bastien P., Wakamatsu K., Bernerd F. (2015). Chemical analysis of constitutive pigmentation of human epidermis reveals constant eumelanin to pheomelanin ratio. Pigment. Cell Melanoma Res..

[7] Hou L., Panthier J.J., Arnheiter H. (2000). Signaling and transcriptional regulation in the neural crest-derived melanocyte lineage: Interactions between KIT and MITF. Development.

[8] D’Mello S.A., Finlay G.J., Baguley B.C., Askarian-Amiri M.E. (2016). Signaling pathways in melanogenesis. Int. J. Mol. Sci..

[9] Liu Z., Wen H., Hailer F., Dong F., Yang Z., Liu T., Han L., Shi F., Hu Y., Zhou J. (2019). Pseudogenization of Mc1r gene associated with transcriptional changes related to melanogenesis explains leucistic phenotypes in Oreonectes cavefish (Cypriniformes, Nemacheilidae). J. Zool. Syst. Evol. Res..

[10] Valverde P., Healy E., Jackson I., Rees J.L., Thody A.J. (1995). Variants of the melanocyte-stimulating hormone receptor gene are associated with red hair and fair skin in humans. Nat. Genet..

[11] Yu S., Wang G., Liao J., Tang M. (2019). Five alternative splicing variants of the TYR gene and their different roles in melanogenesis in the Muchuan black-boned chicken. Br. Poult. Sci..

[12] Guibert S., Girardot M., Leveziel H., Julien R., Oulmouden A. (2004). Pheomelanin coat colour dilution in French cattle breeds is not correlated with the TYR, TYRP1 and DCT transcription levels. Pigment Cell Res..

[13] Yang S.-L., Mao H.-M., Shu W., Deng W.-D. (2006). Melanin traits of Yunnan black bone sheep and TYR gene polymorphism. Hereditas.

[14] Ross E.M., Gilman A.G. (1980). Biochemical properties of hormone-sensitive adenylate cyclase. Annu. Rev. Biochem..

[15] Wootten D., Christopoulos A., Marti-Solano M., Babu M.M., Sexton P.M. (2018). Mechanisms of signalling and biased agonism in G protein-coupled receptors. Nat. Rev. Mol. Cell Biol..

[16] Simon M.I., Strathmann M.P., Gautam N.J.S. (1991). Diversity of G proteins in signal transduction. Science.

[17] Lu Y., Xi J., Zhang Y., Chen W., Zhang F., Li C., Wang Z. (2020). SNHG1 Inhibits ox-LDL-Induced Inflammatory Response and Apoptosis of HUVECs via Up-Regulating GNAI2 and PCBP1. Front. Pharmacol..

[18] Li Z.-W., Sun B., Gong T., Guo S., Zhang J., Wang J., Sugawara A., Jiang M., Yan J., Gurary A.J.G. (2019). GNAI1 and GNAI3 reduce colitis-associated tumorigenesis in mice by blocking IL6 signaling and down-regulating expression of GNAI2. Gastroenterology.

[19] Sun Q., He Q., Xu J., Liu Q., Lu Y., Zhang Z., Xu X., Sun B.J.T.F.J. (2019). Guanine nucleotide–binding protein G (i) α2 aggravates hepatic ischemia-reperfusion injury in mice by regulating MLK3 signaling. FASEB J..

[20] Wang Z., Dela Cruz R., Ji F., Guo S., Zhang J., Wang Y., Feng G.S., Birnbaumer L., Jiang M., Chu W.M. (2014). G(i)α proteins exhibit functional differences in the activation of ERK1/2, Akt and mTORC1 by growth factors in normal and breast cancer cells. Cell Commun. Signal. CCS.

[21] Jung K.S., Kim S.H., Yoon J.T. (2020). Production, Differential Methylation of Melanin-related Epigenetic Genes during Brindle Cattle Growth. J. Anim. Health Prod..

[22] Chen Y., Hu S., Mu L., Zhao B., Wang M., Yang N., Bao G., Zhu C., Wu X. (2019). Slc7a11 Modulated by POU2F1 is Involved in Pigmentation in Rabbit. Int. J. Mol. Sci..

[23] Schmittgen T.D., Livak K.J. (2008). Analyzing real-time PCR data by the comparative C(T) method. Nat. Protoc..

[24] Harris V.M. (2015). Protein Detection by Simple Western™ Analysis. Western Blotting.

[25] Chen T., Zhao B., Liu Y., Wang R., Yang Y., Yang L., Dong C. (2018). MITF-M regulates melanogenesis in mouse melanocytes. J. Dermatol. Sci..

[26] Hu S., Zhai P., Chen Y., Zhao B., Yang N., Wang M., Xiao Y., Bao G., Wu X. (2019). Morphological Characterization and Gene Expression Patterns for Melanin Pigmentation in Rex Rabbit. Biochem. Genet..

[27] Hu S., Chen Y., Zhao B., Yang N., Chen S., Shen J., Bao G., Wu X. (2020). KIT is involved in melanocyte proliferation, apoptosis and melanogenesis in the Rex Rabbit. PeerJ.

[28] Li J., Chen W., Wu S., Ma T., Jiang H., Zhang Q. (2019). Differential expression of MC1R gene in Liaoning Cashmere goats with different coat colors. Anim. Biotechnol..

[29] Zhang J., Li Y., Wu Y., Yang T., Yang K., Wang R., Yang J., Guo H. (2013). Wnt5a inhibits the proliferation and melanogenesis of melanocytes. Int. J. Med Sci..

[30] Su Z., Zheng X., Zhang X., Wang Y., Zhu S., Lu F., Qu J., Hou L.J.J. (2017). Sox10 regulates skin melanocyte proliferation by activating the DNA replication licensing factor MCM5. J. Dermatol. Sci..

[31] Ji K., Zhang P., Zhang J., Fan R., Liu Y., Yang S., Hu S., Liu X., Dong C. (2018). MicroRNA 143-5p regulates alpaca melanocyte migration, proliferation and melanogenesis. Exp. Dermatol..

[32] Wang Y., Lan Y., Lu H. (2020). Opsin3 Downregulation Induces Apoptosis of Human Epidermal Melanocytes via Mitochondrial Pathway. Photochem. Photobiol..

